# A country bug in the city: urban infestation by the Chagas disease vector *Triatoma infestans* in Arequipa, Peru

**DOI:** 10.1186/1476-072X-12-48

**Published:** 2013-10-30

**Authors:** Stephen Delgado, Kacey C Ernst, María Luz Hancco Pumahuanca, Stephen R Yool, Andrew C Comrie, Charles R Sterling, Robert H Gilman, César Náquira, Michael Z Levy

**Affiliations:** 1 School of Geography and Development, The University of Arizona, 409 Harvill Building, 1103 East Second Street, Tucson, Arizona 85721, USA; 2 Division of Epidemiology and Biostatistics, The University of Arizona, Roy P Drachman Hall, 1295 North Martin Avenue, PO Box 245211, Tucson, Arizona 85724, USA; 3 Facultad de Ciencias y Filosofia, Universidad Peruana Cayetano Heredia, Avenida Honorio Delgado 430, Urbanización Ingeniería, Lima, Peru; 4 School of Animal and Comparative Biomedical Sciences, The University of Arizona, 1117 East Lowell Street, Tucson, Arizona 85721, USA; 5 Bloomberg School of Public Health, Johns Hopkins University, 615 North Wolfe Street, Baltimore, Maryland 21205, USA; 6 Department of Biostatistics and Epidemiology, University of Pennsylvania, 714 Blockley Hall, 423 Guardian Drive, Philadelphia, Pennsylvania 19104, USA

**Keywords:** *Triatoma infestans*, Chagas disease, Urban infestation, Vector control, Spatial analysis, Multilevel logistic regression

## Abstract

**Background:**

Interruption of vector-borne transmission of *Trypanosoma cruzi* remains an unrealized objective in many Latin American countries. The task of vector control is complicated by the emergence of vector insects in urban areas.

**Methods:**

Utilizing data from a large-scale vector control program in Arequipa, Peru, we explored the spatial patterns of infestation by *Triatoma infestans* in an urban and peri-urban landscape. Multilevel logistic regression was utilized to assess the associations between household infestation and household- and locality-level socio-environmental measures.

**Results:**

Of 37,229 households inspected for infestation, 6,982 (18.8%; 95% CI: 18.4 – 19.2%) were infested by *T. infestans*. Eighty clusters of infestation were identified, ranging in area from 0.1 to 68.7 hectares and containing as few as one and as many as 1,139 infested households. Spatial dependence between infested households was significant at distances up to 2,000 meters. Household *T. infestans* infestation was associated with household- and locality-level factors, including housing density, elevation, land surface temperature, and locality type.

**Conclusions:**

High levels of *T. infestans* infestation, characterized by spatial heterogeneity, were found across extensive urban and peri-urban areas prior to vector control. Several environmental and social factors, which may directly or indirectly influence the biology and behavior of *T. infestans*, were associated with infestation. Spatial clustering of infestation in the urban context may both challenge and inform surveillance and control of vector reemergence after insecticide intervention.

## Background

Chagas disease, also known as American trypanosomiasis, is caused by the protozoan parasite *Trypanosoma cruzi* and is endemic in Latin America. Typically, *T. cruzi* is transmitted to humans via the infected excretions of various blood-feeding triatomine insect species, including *Triatoma infestans*. Less commonly, infection may result from congenital transmission, blood transfusion, organ transplantation, and incidental ingestion of parasite-contaminated food or drink [1]. Chagas disease is characterized by an acute phase, which lasts 6 – 8 weeks, and a chronic phase, which persists for life. In most cases, both the acute and chronic phases of infection are asymptomatic. However, 10 – 40% of cases, depending on the geographic region, progress over a period of years to chronic disease, including potentially fatal cardiac and gastrointestinal disorders [2]. Globally, eight million persons are infected with *T. cruzi*, resulting in 11,000 deaths and the loss of 430,000 disability-adjusted life years (DALYs) annually [3,4].

Prevention and control of Chagas disease are achieved primarily via large-scale insecticide application initiatives [5], and *T. infestans*, which lives predominantly in and around human households, is a principal target for vector control [2]. While the Southern Cone Initiative has succeeded in interrupting *T. cruzi* transmission by *T. infestans* in Brazil, Chile, and Uruguay [6,7], *T. infestans*-mediated *T. cruzi* transmission persists in parts of Argentina, Bolivia, Paraguay, and Peru [8,9]. Moreover, efforts to eliminate *T. infestans* have been complicated by the expansion of this species from sparsely populated rural regions into densely populated urban areas [10,11].

While the ecology of *T. infestans* infestation in rural environments has been studied extensively, urban infestation by this insect has been investigated only preliminarily. To advance understanding of vector infestation in the urban context, this study examines infestation by *T. infestans* across an urban and peri-urban landscape prior to implementation of vector control. Our study focuses on Arequipa, Peru, which lies in an area with extensive *T. infestans* infestation and epidemic *T. cruzi* transmission [8,12]. Utilizing data collected from multiple sources at multiple spatial scales, we employ spatial point pattern analysis and multilevel logistic regression to elucidate spatial patterns in infestation by *T. infestans* and to assess associations between several environmental and social factors and *T. infestans* infestation in an urban landscape. In particular, the effects of housing density, land surface temperature, and elevation were evaluated at the household level, while the effects of urban shantytowns, which have been identified as areas at higher risk for infestation by Chagas diesease vectors and vector-borne transmission of *T. cruzi*, were evaluated at the locality level [11,13-15].

## Results

**Figure 1:**
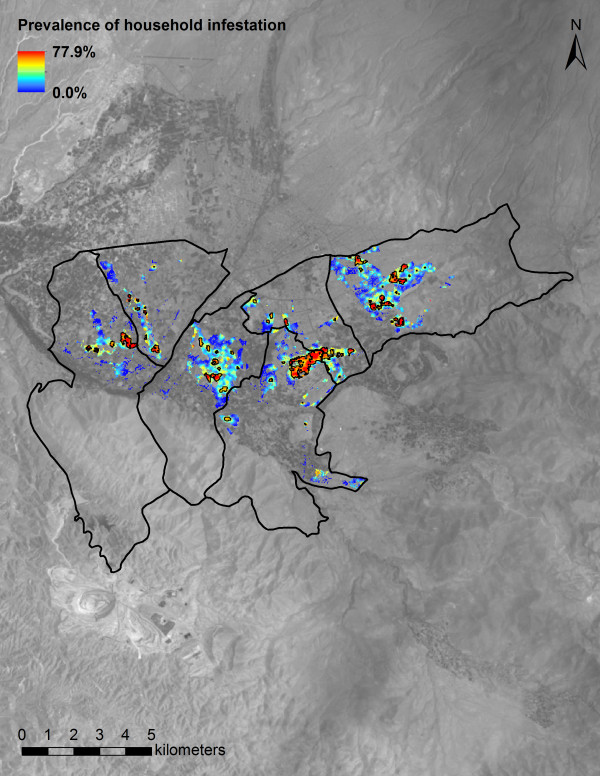
**Kernel-smoothed prevalence of household *Triatoma infestans *infestation.** Spatially smoothed prevalence of household infestation varied from 0.0 – 77.9% across the study area. Colored pixels outlined in black represent statistically significant clusters of infestation. A grayscale Landsat 5 Thematic Mapper (TM) band 3 image (15 December 2008, WGS 84 UTM 19S) shows landscapes encompassed by the six study area districts and surrounding areas.

*Triatoma infestans* were found in 6,982 (18.8%; 95% CI: 18.4 – 19.2%) of the 37,229 study households. Prevalence of household infestation varied widely across the study area, with spatially smoothed estimates ranging from 0.0 to 77.9% (Figure 1). Eighty areas were identified that exceeded the upper limit of a 999-iteration random labeling simulation of the kernel density estimate ratio of infested households versus all study households. These clusters of infestation, which ranged in area from 0.1 to 68.7 hectares, contained as few as 1 and as many as 1,139 infested households. In total, the clusters encompassed 3,278 (46.9%) of the 6,982 infested households. The K-function difference for infested versus non-infested households exceeded the upper limit of a 999-iteration random labeling simulation at all 100-meter increments up to 2000 meters.

Univariate logistic regression showed statistically significant relationships between household infestation and housing density, elevation, and land surface temperature. No significant correlation was found between housing density and elevation (r = 0.13), between housing density and land surface temperature (r = 0.02), or between elevation and land surface temperature (r = 0.02). For housing density, odds of infestation were approximately two times higher in the highest versus the lowest quintile. For elevation, odds of infestation were higher only in the third and fourth quintiles. Odds of infestation increased 8% with each 1°C increase in land surface temperature. Covariates remained statistically significant in the multivariate model, with a modest increase in the odds of infestation associated with land surface temperature, and moderate decreases in the odds of infestation associated with housing density and elevation (Table 1).

**Table 1:** Results of univariate and multivariate logistic regression

			***Univariate logistic regression***		***Multivariate logistic regression***	
**Range**	**Inspected**	**Infested**	**Odds ratio**	**95% CI**	**Odds ratio**	**95% CI**
*Housing density (households/hectare)*				AIC = 32,993		
2 – 24*	6,866	831	1.00		1.00	
24 – 30	6,855	1,148	1.46	1.33 – 1.61	1.38	1.26 – 1.53
30 – 34	6,848	1,434	1.92	1.75 – 2.11	1.79	1.63 – 1.97
34 – 39	6,861	1,582	2.18	1.99 – 2.39	2.02	1.84 – 2.22
39 – 77	6,845	1,534	2.10	1.91 – 2.30	1.90	1.73 – 2.09
*Land surface temperature (°C)*				AIC = 33,300		
26 – 40	34,275	6,529	1.08	1.06 – 1.10	1.10	1.08 – 1.12
*Elevation (meters above sea level)*				AIC = 33,134		
2,120 – 2,260*	6,866	1,085	1.00		1.00	
2,260 – 2,300	6,996	1,089	0.98	0.90 – 1.08	0.82	0.74 – 0.90
2,300 – 2,350	6,716	1,526	1.57	1.44 – 1.71	1.31	1.20 – 1.44
2,350 – 2,450	6,852	1,612	1.64	1.50 – 1.79	1.45	1.33 – 1.59
2,450 – 2,670	6,845	1,217	1.15	1.05 – 1.26	1.06	0 .96 – 1.16
						AIC = 32,724

*Referent category.

95% CI: 95% confidence interval.

AIC: Akaike information criteria.

Multilevel logistic regression represented an improvement over ordinary logistic regression, and the data were best fit by a model including household-level covariates, a locality-level covariate, and locality-level random effects (Table 2).

**Table 2:** Results of multilevel logistic regression

	***Model 0***	***Model 1***	***Model 2***
**Household-level fixed effects: odds ratio (95% CI)**			
*Housing density (households/hectare)*			
2 – 24*		1.00	1.00
24 – 30		1.23 (1.10 – 1.37)	1.22 (1.10 – 1.37)
30 – 34		1.44 (1.29 – 1.61)	1.44 (1.29 – 1.61)
34 – 39		1.57 (1.39 – 1.76)	1.56 (1.39 – 1.75)
39 – 77		1.74 (1.54 – 1.97)	1.73 (1.53 – 1.96)
*Land surface temperature (°C)*			
26 – 40		1.10 (1.08 – 1.13)	1.10 (1.08 – 1.13)
*Elevation (meters above sea level)*			
2120 – 2260*		1.00	1.00
2260 – 2300		1.44 (1.18 – 1.76)	1.48 (1.21 – 1.82)
2300 – 2350		1.89 (1.49 – 2.41)	1.99 (1.57 – 2.53)
2350 – 2450		2.12 (1.63 – 2.76)	2.28 (1.75 – 2.98)
2450 – 2670		1.39 (1.07 – 1.82)	1.49 (1.14 – 1.94)
**Locality-level fixed effects: odds ratio (95% CI)**			
Shantytown			1.75 (1.24 – 2.47)
			IOR: (0.30 – 10.26)
**Locality-level random effects**			
Variance (95% CI)	1.12 (0.84 – 1.49)	1.03 (0.77 – 1.37)	0.95 (0.71 – 1.27)
Change in variance		−8.1%	−7.4%
Median odds ratio	2.74	2.63	2.54
**Likelihood ratio test (p-value)**			
v. logistic model^†^	< 0.0001	< 0.0001	< 0.0001
v. multilevel model^‡^		< 0.0001	0.0015
AIC	30,467	30,269	30,261

*Referent category.

^†^Multilevel versus ordinary logistic regression.

^‡^Multilevel model n + 1 versus model n.

95% CI: 95% confidence interval.

IOR: interval odds ratio.

AIC: Akaike information criteria.

**Figure 2:**
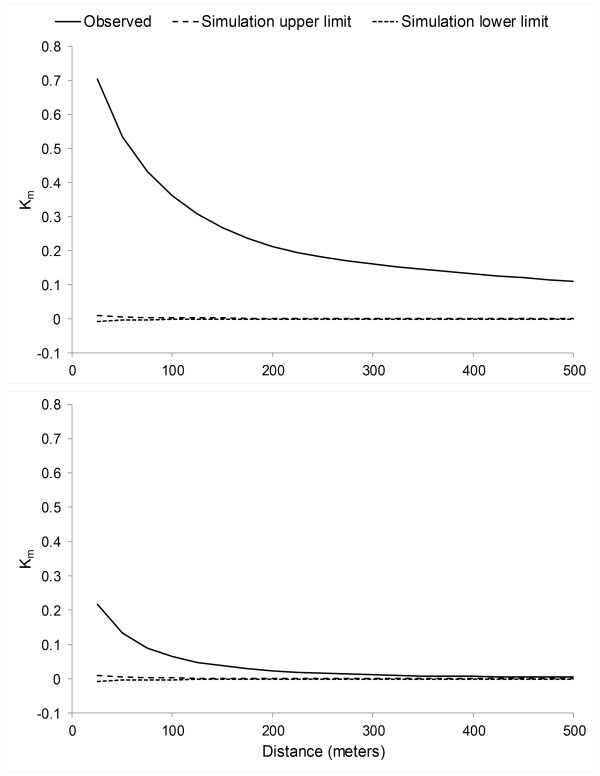
**Spatial autocorrelation in deviance residuals for least- and best-fit regression models.** Comparison of spatial autocorrelation in the deviance residuals from the ordinary multivariate logistic regression model (upper panel) versus the best-fit multilevel logistic regression model (lower panel). The mark correlation function (K_m_) may vary between −1 (negative spatial autocorrelation) and +1 (positive spatial autocorrelation), with an expected value of 0 for no spatial autocorrelation. Spatial autocorrelation was substantially reduced, albeit not eliminated, in the best- versus least-fit multivariate logistic regression model.

Spatial autocorrelation in deviance residuals was distinctly decreased in the best-fit multilevel model versus the ordinary multivariate model (Figure 2).

Locality-level random effects were substantial. The median odds ratio (MOR), which is the median value of the odds ratio when comparing a higher to a lower risk locality, indicated that the median odds of infestation were two and one-half times greater in higher versus lower risk localities (Table 2).

The effect of the locality-level covariate, locality type, was also significant. Households located in shantytowns had 75% higher odds of infestation than households situated in other locality types. However, the interval odds ratio (IOR), which is the interval between the 10th and 80th percentile centered on the median value of the distribution of odds ratios for locality type, included the value one. This indicates that the effect of locality type is not as strong as the locality-level random effect (Table 2).

Household-level effects all remained statistically significant. Odds of infestation increased 10% with each 1 °C increase in land surface temperature, which was similar to the estimate from ordinary multivariate logistic regression. The highest housing density quintile had 75% higher odds of infestation relative to the lowest quintile, which is slightly diminished compared to results from the ordinary multivariate model. The fourth elevation quintile had greater than twice the odds of infestation relative to the lowest quintile, but the highest quintile showed only 50% higher odds of infestation relative to the lowest quintile. The effect of elevation was substantially increased in the multilevel versus the ordinary multivariate logistic regression model (Table 2).

## Discussion

Infestation by *T. infestans* has been found in many urban areas in Latin America, including Santiago, Chile [13]; Cochabamba and Sucre, Bolivia [14]; and Arequipa, Peru [11]. In affected areas—urban as well as rural—prevention and control of Chagas disease relies on vector control [5]. While infestation by and control of *T. infestans* has been extensively examined in the rural context, infestation in the urban milieu is less well understood. Utilizing spatial and multilevel logistic regression analysis of data collected from multiple sources at multiple spatial scales, we offer insights into the dynamics of *T. infestans* infestation in an urban landscape.

Prior to implementation of vector control, urban and peri-urban households in Arequipa were extensively infested by *T. infestans*. The intensity of infestation was spatially heterogeneous, with areas of very low and very high prevalence of infestation. Numerous clusters of infestation, small and large, were found across the six study districts, indicating that urban and peri-urban areas are conducive to the proliferation and dispersion of *T. infestans*. In rural landscapes, *T. infestans* have been shown to actively disperse by walking or flying at distances up to approximately 100 or 2,000 meters, respectively [16,17]. In a separate study in urban Arrequipa, streets were shown to be significant barriers to the dispersion of *T. infestans*, and to strongly influence the spatial distribution of infestation [18]. In contrast, flight has been observed as a main mechanism of infestation in urban San Juan, Argentina [19]. In the present study, spatial dependence between infested households was observed at distances from 0 to 2,000 meters, suggesting that urban *T. infestans* may disperse by walking at shorter spans that do not cross city streets, as well as by flying at longer distances across urban blocks.

Identifying extant clusters of infestation prior to vector control may have critical consequences for implementing effective surveillance of vector reemergence subsequent to vector control. In an extensive but sparsely populated rural area in the Gran Chaco of Argentina, reinfestation by *T. infestans* tended to cluster in areas where infestation was aggregated prior to vector control [20]. Infestation clusters in an extensively and densely populated urban area may be similarly problematic. The existence of numerous infestation clusters in Arequipa, many encompassing large areas and many households, should be priority areas for surveillance and control by the GRSA. Where feasible, utilization of a geographic information system to monitor *T. infestans* reemergence—as well as other health risks and outcomes—might be a cost effective investment for resource-constrained public health institutions in Arequipa, and elsewhere in the developing world [21,22].

Spatial heterogeneity in urban infestation by *T. infestans* is likely influenced by myriad factors operating at multiple spatial scales. We evaluated only a few features, which were chosen based on ecological plausibility and data availability. In ordinary univariate and multivariate logistic regression, housing density, elevation, and land surface temperature were all positively, if not always linearly, associated with household infestation. Housing density may mediate vector dispersal. In higher density urban areas, new habitats and blood sources found in nearby houses are located at short distances from one another, thereby facilitating dispersal of refuge- or blood-seeking vectors. Also, attraction to light influences the dissemination of *T. infestans*, and the plentiful light sources in higher density urban areas may promote insect dispersal [23]. Land surface temperature may affect vector biology and behavior. Both laboratory and field experiments demonstrate that *T. infestans* flight initiation increases at higher temperatures [17,24], thereby promoting vector dispersal in warmer urban areas. Laboratory studies also indicate that higher temperatures increase *T. infestans* feeding and development rates [25,26], and blood meal seeking is reportedly the principal cause for dispersion of triatomines [23]. In warmer urban areas, increased feeding and development may result in increased vector dispersal. Elevation may act indirectly through socioeconomic circumstances, rather than directly through biophysical constraints. In Arequipa, lower socioeconomic status populations, often rural-to-urban migrants, typically inhabit the higher elevation hillsides, while higher socioeconomic status populations usually reside in lower elevation valleys [27]. As such, higher infestation at higher elevation in Arequipa may be attributable to two factors: passive introduction of insects resulting from seasonal migration to and from nearby rural areas where *T. infestans* are prevalent, and substandard living conditions that provide habitats suitable for *T. infestans* infestation [27]. The slight decrease in infestation at the highest elevations may result from the relatively recent inhabitation of these areas, leaving little time for infestation to have occurred. Elevation is unlikely to be a biophysical constraint for infestation in the currently populated areas of Arequipa, since *T. infestans* have been found as high as 3,682 meters above sea level in Argentina [23], well above the elevation of the study area.

Multilevel logistic regression revealed the importance of locality-level contextual effects and substantially diminished spatial autocorrelation present in ordinary logistic regression. The locality-level random effect, which estimates the influence of unobserved contextual effects within each locality, indicates that these unmeasured factors are associated, in median, with substantially higher risk of household infestation. In Arequipa, and elsewhere, urban shantytowns have been identified as areas with higher risk for infestation by Chagas disease vectors, and vector-borne transmission of *T. cruzi*[11,13-15]. We offer further evidence that shantytowns are at higher risk for infestation by *T. infestans*. Controlling for locality-level effects, household-level effects for housing density, elevation, and land surface temperature all remained statistically significant and substantial.

We recognize that our study is limited in many respects. First, while we believe that household location and infestation status data are both precise and accurate, more detailed data regarding the number, life stage, and *T. cruzi* infection status of insects encountered during the vector control campaign were unavailable. Nor did we have in-depth data regarding households (e.g., construction materials, domestic animals) or their occupants. More detailed data would have likely improved the insights provided by our analyses. Second, we recognize that point-level household covariates are extracted from remote sensing data collected at a 30-meter scale (elevation, land surface temperature) or are spatially smoothed estimates (housing density). We also understand that land surface temperature data do not capture fine-scale temporal variability that occurs across and within days of the year, nor do they describe fine-scale spatial variation in ambient micro-climatic conditions. These issues of scale could conceivably bias the relative magnitude of observed effects. Third, at the time of data collection, portions of the six study districts were still undergoing vector control. Future analyses of areas recently reached by the vector control campaign, including districts beyond the current study area, may provide deeper and broader insights into urban and peri-urban *T. infestans* infestation.

The geography and ecology of *T. infestans*—as well as vector species for many other infectious diseases—are changing. Decreasing funding and political will and increasing insecticide resistance are endangering gains made towards interruption of vector-borne transmission of *T. cruzi*[9]. For many vector-borne diseases in many parts of the world, these are not only public health concerns but also social justice issues, as economically and politically marginalized populations may suffer disproportionately. Many potentially powerful tools (e.g., Google Earth, The R Project for Statistical Computing), data sources (e.g., NASA, NOAA), and spatial and statistical methods are now freely available. Finding novel uses for these resources in conjunction with local knowledge and information—as well as increasing capacity to do so—could inspire new perspectives on and solutions to existing and emerging public health problems and their social and environmental causes and consequences.

## Methods

### Study area

**Figure 3:**
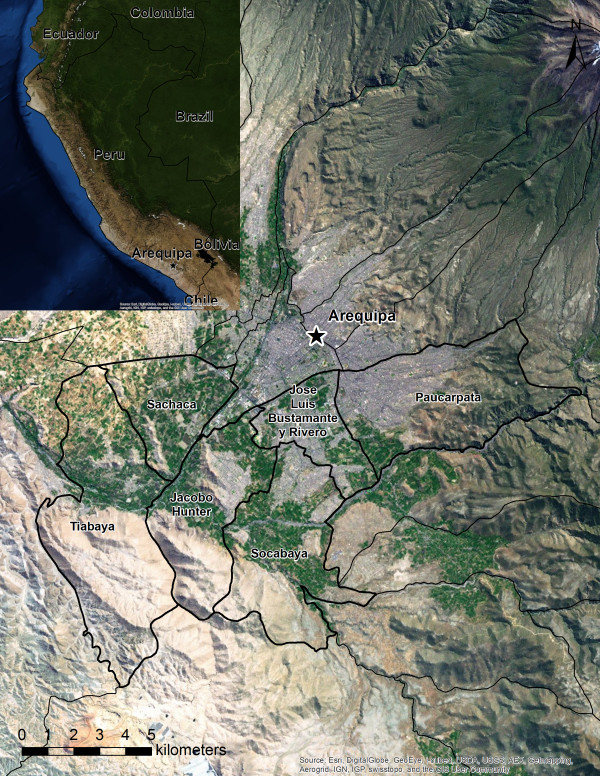
**The Arequipa, Peru, study area.** Satellite imagery of the six study area districts, the city of Arequipa, and the surrounding area. The inset map shows the locations of Arequipa, Peru, and bordering areas. The study area encompasses a mosaic of urban and peri-urban landscapes.

Arequipa (population 864,250) is situated in southwestern Peru and is the country’s third most populous province. This study focuses on six of the province’s twenty-nine districts: Jacobo Hunter, Jose Luis Bustamante y Rivero, Paucarpata, Sachaca, Socabaya, and Tiabaya. The districts encompass a geographically contiguous area of 139 square kilometers adjacent to the capital city of Arequipa, and include nearly forty percent of the province’s population [28] (Figure 3).

In 2003, the *Gerencia Regional de Salud de Arequipa* (GRSA) initiated a vector control program to eliminate household infestation by *T. infestans*. The program is ongoing, is implemented on a district-by-district basis, and consists of a simple stepwise process. First, each household is assigned a unique alphanumeric code, and household codes and locations are catalogued on hand-drawn maps. Second, trained GRSA personnel go door-to-door, spray all domestic areas and peri-domestic animal enclosures in each household, and inspect for the presence of triatomines, taking advantage of the flushing effect of the insecticide. The presence or absence of *T. infestans* is recorded. Finally, the inspection and insecticide application process is repeated approximately six months later.

### Data collection

#### Household data

Maps of household locations, household unique identifier codes, and dates and results of household *T. infestans* inspections were provided by the GRSA. Within the study area 37,229 households had been inspected for infestation by *T. infestans* and sprayed with insecticide during the period from September 2003 through December 2008. Using GRSA maps and Google Earth imagery, we assigned geographic coordinates to these households, as well as to households within the six study districts that had not as yet been reached by the vector control campaign as of December 2008. Household geographic coordinates, household *T. infestans* infestation status (0/1), and household unique identifier codes were stored in a relational database management system for subsequent analysis.

#### Remote sensing data

Advanced Spaceborne Thermal Admission and Reflection Radiometer (ASTER) Global Digital Elevation Model Version 2 (GDEM V2) 30-meter-resolution imagery of the study area (ASTGTM2_S17W072) was acquired from the National Aeronautics and Space Administration (NASA) Earth Observing System Data and Information System (EOSDIS) [29]. Landsat 5 Thematic Mapper (TM) 120-meter resolution thermal imagery (band 6: 10.40 – 12.50 μm) and 30-meter resolution visible (band 3: 0.63 – 0.69 μm) and near-infrared (band 4: 0.76 – 0.90 μm) imagery of the study area (WRS path 3 row 71) were obtained from the United States Geological Survey (USGS) EarthExplorer [30]. Cloud-free images were attained for nine dates in 2008: 18 March, 19 April, 21 May, 24 July, 25 August, 26 September, 12 October, 13 November, and 15 December. A Landsat 5 TM band 3 image acquired on 25 July 1987 was obtained from NASA’s Global Orthorectifed Landsat Data Set as reference for geometric correction of 2008 images [31,32].

#### Census data

In Peru, census areas are subdivided into departments, provinces, districts, and localities. A database of the locality in which each study household was located was provided by the GRSA, and a census classification of locality types was obtained from the Peru National Institute of Statistics and Informatics (INEI). Locality type categories included city (*ciudad*), housing development (*urbanización*), town (*pueblo*), shantytown (*pueblo joven*), housing association (*asociación de viviendas*), housing cooperative (*cooperativa de viviendas*), annex (*anexo*), hamlet (*caserío*), and rural community (*comunidad campesina*) [33]. The GRSA and INEI databases were joined and each household was assigned a categorical variable specifying locality type for 34,275 of the 37,229 (92.1%) households with documented *T. infestans* inspection data.

### Data analysis

#### Spatial point pattern analysis

Spatial variation in household infestation by *T. infestans* was evaluated by dividing the kernel-smoothed density of infested households by the kernel-smoothed density of all study households. An isotropic Gaussian smoothing kernel with a standard deviation (σ) of 45.7 meters was utilized for this analysis, where σ was selected using a likelihood cross-validation method. A 999-iteration random labeling simulation was performed to identify areas where infestation by *T. infestans* was significantly elevated [34,35]. The kernel-smoothed density of all georeferenced households (n = 68,849), with σ = 27.8 meters, was estimated for evaluation as a covariate in logistic regression [35,36].

Spatial dependence between households infested by *T. infestans* was assessed by computing the difference in the K-function for infested households and non-infested households at 100-meter increments from 0 to 2000 meters. A 999-iteration random labeling simulation was executed to identify distances at which spatial dependence of infested households was statistically significant [37].

Spatial statistical analyses were conducted using R (The R Project for Statistical Computing) [38]. Maps of spatial variation in household infestation and household density were constructed using ArcGIS version 10 (ESRI) [39].

#### Remote sensing image analysis

Remote sensing data were utilized to derive and extract household point-level estimates of elevation and land surface temperature for evaluation as covariates in logistic regression.

The ASTER GDEM V2 image was projected to the WGS 84 UTM 19S coordinate system and resampled to a 30-meter pixel size to match the projection and spatial resolution of Landsat 5 TM imagery, and elevation data were extracted to household point locations.

Landsat 5 TM and ASTER GDEM V2 images were cropped to a 701-column by 474-row area corresponding to the rectangle bounding the six study area districts. Landsat 5 TM band 3, 4, and 6 images from 2008 were geometrically corrected utilizing a simple root mean square error minimization routine and the Landsat 5 TM band 3 image from 1985 as reference [40]. Atmospheric correction of Landsat 5 TM band 3 and 4 images was performed using a modified dark object subtraction method [41], followed by topographic correction utilizing a Minnaert method [42]. The normalized difference vegetation index (NDVI) was calculated from Landsat 5 TM band 3 and 4 images [43], and land surface emissivity was estimated from NDVI for each date in 2008 [44]. Land surface temperature was derived using Landsat 5 TM band 6 thermal infrared images; land surface emissivity images; and coefficients for atmospheric transmissivity, upwelling atmospheric radiance, and downwelling atmospheric radiance [45,46]. Land surface temperature images were overlaid, median land surface temperature was calculated for each raster pixel, and these data were extracted to household point locations.

Processing of ASTER GDEM V2 and Landsat 5 TM images was conducted using the landsat package version 1.0.8 in R version 2.15.2 [40]. Atmospheric coefficients were obtained from the Atmospheric Correction Parameter Calculator [47]. ArcGIS version 10 was utilized to create maps of elevation and land surface temperature, and to extract values for these variables to household point locations.

#### Statistical analysis

**Figure 4:**
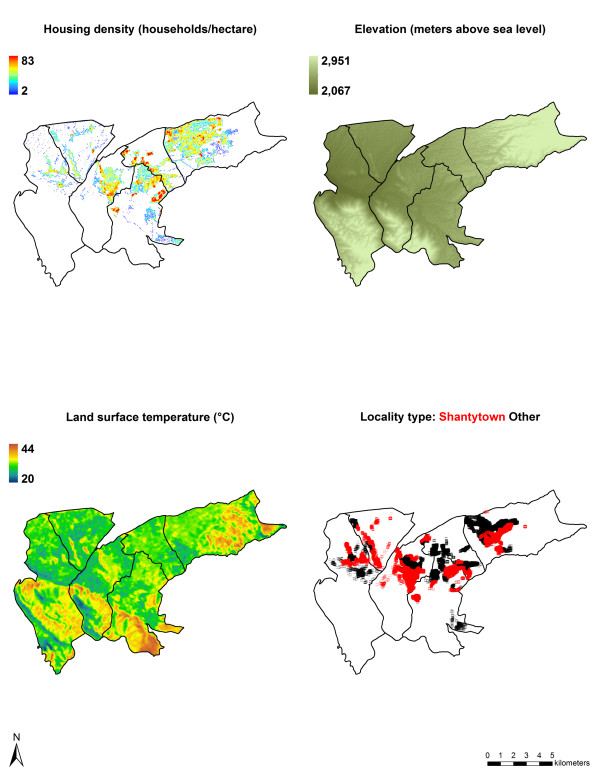
**Household- and locality-level variables associated with *Triatoma infestans *infestation.** Spatial distributions of three household-level variables (housing density, elevation, and land surface temperature) and one locality-level variable (locality type) that were evaluated in logistic regression modeling. These socio-environmental variables, each of which influences urban *T. infestans* infestation, exhibit distinct spatial variation across the study area.

Prevalence of infestation was calculated for the 37,229 households inspected and sprayed for *T. infestans*. Pearson’s correlation coefficient was utilized to assess correlation among candidate continuous covariates for logistic regression modeling. Univariate and multivariate logistic regression were used to evaluate the associations between household infestation and household-level variables, including housing density, elevation, and median land surface temperature (Figure 4). To account for non-linearity in logistic regression, housing density and elevation were converted from continuous to categorical variables based on their respective quintiles. Median land surface temperature was maintained as a continuous variable.

To address spatial dependence among observations and to assess locality-level effects, three multilevel logistic regression models were evaluated: a model including only locality-level random effects (Model 0); a model including household-level covariates and locality-level random effects (Model 1); and a model including household-level covariates, a locality-level covariate, and locality-level random effects (Model 2). In addition to standard odds ratios, median odds ratios (MOR) were calculated for locality-level random effects, and the interval odds ratio (IOR) were calculated for the locality-level covariate [48,49].

The locality-level covariate is a dichotomous categorization of locality type into shantytown and other. Shantytowns are informal urban population centers composed of blocks or collection of substandard housing, often without urban infrastructure or basic services [33]. In contrast, the category other consists primarily of formal urban population centers, including cities, housing developments, housing associations, and housing cooperatives; and secondarily of formal rural population centers, including towns, hamlets, rural communities, and annexes [33]. In Peru, urban areas are defined as built areas of at least 100 households that are continuously occupied, whereas rural areas are simply defined as built areas outside of urban areas [33]. Among urban locality types, cities correspond to common conceptions of cities, housing developments resemble suburbs, housing associations are residential housing developments with shared living spaces, and housing cooperatives are residential housing developments with shared living spaces. Among rural locality types, towns correspond to common conceptions of rural towns, hamlets are smaller versions of towns, rural communities are communal farming areas, and annexes correspond to unincorporated areas. Locality type information was unavailable for 2,954 (7.9%) of mapped households. These were omitted from logistic regression modeling, leaving 34,725 households located in 160 localities for regression analyses (Table 3).

**Table 3:** Categorization scheme for locality type

***Category***	***Locality type***	***Households***		***Localities***	
		**Number**	**Percent**	**Number**	**Percent**
*Shantytown*	Shantytown	16,595	44.58	87	40.28
*Other*	Housing development	13,293	35.71	51	23.61
	City	2,308	6.20	2	0.93
	Town	1,009	2.71	5	2.31
	Hamlet	567	1.52	5	2.31
	Housing cooperative	354	0.95	5	2.31
	Annex	82	0.22	3	1.39
	Rural community	52	0.14	1	0.46
	Housing association	15	0.04	1	0.46
*No data*	No data	2,954	7.93	56	25.93
		37,229	100.00	216	100.00

Locality type was categorized into shantytown (n = 16,595 households in 87 localities) and other (n = 17,680 households in 73 localities). The 2,954 households without locality type data were omitted from logistic regression analyses.

Model 0 Locality-level random effects only:

$$\mathrm{logit}\left[\mathrm{Pr}\left(y_{\mathrm{ij}}=1\right)\right]=\beta_{0}+u_{j}$$

Model 1 Household-level effects and locality-level random effects:

$$\mathrm{logit}\left[\mathrm{Pr}\left(y_{\mathrm{ij}}=1\right)\right]=\beta_{0}+\beta_{1}\;\mathrm{densit}y_{\mathrm{ij}}+\beta_{2}\;\mathrm{elevatio}n_{\mathrm{ij}}+\beta_{3}\;\mathrm{temperatur}e_{\mathrm{ij}}+u_{j}$$

Model 2 Household- and locality-level effects and locality-level random effects:

$$\mathrm{logit}\left[\mathrm{Pr}\left(y_{\mathrm{ij}}=1\right)\right]=\beta_{0}+\beta_{1}\;\mathrm{densit}y_{\mathrm{ij}}+\beta_{2}\;\mathrm{elevatio}n_{\mathrm{ij}}+\beta_{3}\;\mathrm{temperatur}e_{\mathrm{ij}}+\beta_{4}\;\mathrm{locality}\;\mathrm{typ}e_{j}+u_{j}$$

Logit is the link function; Pr(*y*_*ij*_ = 1) is the probability of household infestation; *i* and *j* indicate the *i*th household and the *j*th locality, *β*_*1*_ is the vector of regression coefficients for *density*, where *density* is kernel-smoothed housing density (households/hectare) categorized by quintiles; *β*_*2*_ is the vector of regression coefficients for *elevation*, where *elevation* (meters above sea level) is categorized by quintiles; *β*_*3*_ is the regression coefficient for *temperature*, where *temperature* is estimated annual median land surface temperature (°C); *β*_*4*_ is the regression coefficient for *locality type*, where *locality type* is a dichotomous categorization of localities into shantytown and other; *β*_*0*_ is the household-level intercept; and *u*_*j*_ is the locality-level random effect.

Regression model goodness of fit was assessed using the likelihood ratio test and the Akaike information criteria (AIC). Spatial autocorrelation in deviance residuals of the least- and best-fit models was evaluated utilizing the mark correlation function (K_m_) at 25-meter increments from 0 to 500 meters. A 999-iteration random labeling simulation was executed to identify distances at which regression residual spatial autocorrelation was statistically significant [50].

Stata/IC 12.1 was utilized for statistical analyses [51], and R version 2.15.2 was used for spatial analysis of regression residuals.

## Competing interests

The authors declare that they have no competing interests.

## Authors’ contributions

SD had primary responsibility for data collection and data analysis, and he wrote the manuscript. KCE guided statistical analysis and manuscript revisions. MLHP provided invaluable assistance with data collection and database management. SRY gave expert guidance on remote sensing analyses. ACC provided counsel on data analysis and manuscript preparation. CRS offered authoritative advice on vector biology and behavior and interpretation of results. As principal investigators for the NIH-funded project in which this study is nested, RHG and CN were responsible for overall study design. As project leader, MZL was integrally involved in data collection, data analysis, interpretation of results, and manuscript preparation. All authors have read and approved this manuscript.
